# Bronchial rupture following endobronchial blocker placement: a case report of a rare, unfortunate complication

**DOI:** 10.1186/s12871-021-01430-6

**Published:** 2021-08-30

**Authors:** Shuwen Oo, Rachel Hui Xuan Chia, Yue Li, Hari Kumar Sampath, Sophia Bee Leng Ang, Suresh Paranjothy, John Kit Chung Tam, Chang Chuan Melvin Lee

**Affiliations:** 1 Department of Anaesthesia, National University Health System, Singapore, Singapore; 2 Department of Cardiothoracic and Vascular Surgery, National University Health System, Singapore, Singapore; 3 Department of Cardiothoracic and Vascular Surgery, National University Heart Centre, Singapore, Singapore

**Keywords:** Bronchial blocker, Bronchial rupture, Bronchial injury, Bronchi, Thoracic surgery, Intubation, Airway trauma, Lung separation

## Abstract

**Background:**

Lung separation may be achieved through the use of double lumen tubes or endobronchial blockers. The use of lung separation techniques carries the risk of airway injuries which range from minor complications like postoperative hoarseness and sore throat to rare and potentially devastating tracheobronchial mucosal injuries like bronchus perforation or rupture. With few case reports to date, bronchial rupture with the use of endobronchial blockers is indeed an overlooked complication.

**Case presentation:**

A 78-year-old male patient with a left upper lobe lung adenocarcinoma underwent a left upper lobectomy with a Fuji Uniblocker® as the lung separation device. Despite an atraumatic insertion and endobronchial blocker balloon volume within manufacturer specifications, an intraoperative air leak developed, and the patient was found to have sustained a left mainstem bronchus rupture which was successfully repaired and the patient extubated uneventfully. Unfortunately, the patient passed on in-hospital from sepsis and other complications.

**Conclusion:**

Bronchial rupture is a serious complication of endobronchial blocker use that can carry significant morbidity, and due care should be exercised in its use and placement. Bronchoscopy should be used during insertion, and the volume and pressure of the balloon kept to the minimum required to prevent air leak. Bronchial injury should be considered as a differential in the presence of an unexplained air leak.

**Supplementary Information:**

The online version contains supplementary material available at 10.1186/s12871-021-01430-6.

## Background

Lung separation is a technique employed to facilitate exposure in thoracic surgical procedures, including minimally invasive cardiac, lung, and esophageal surgery. Its indications also extend to control of ventilation distribution, and prevention of cross-contamination of healthy lung by blood or infectious material. This is commonly achieved by insertion of either double lumen tubes (DLTs) or endobronchial blockers (EBBs). Inserted through a single lumen tube (SLT), EBBs may be advantageous in patients with a difficult airway, and reduce the need for tube exchange in patients with a pre-existing SLT in-situ, or those expected to remain intubated in the intensive care unit. Endobronchial blockers may also reduce the incidence of postoperative hoarseness, sore throat, and vocal cord lesions when compared to DLTs [1]. Although tracheobronchial mucosal injury can occur with the use of EBBs, bronchus perforation or rupture is rare, and few case reports exist in the literature [2]. Until now, bronchial and tracheal rupture has been more frequently reported with DLT use as opposed to EBBs. We present an unfortunate case of intraoperative left mainstem bronchus rupture in a patient who underwent left upper lobectomy using a Fuji Uniblocker® (Fuji Systems Corporation, Japan) (supplementary image) for lung separation, which has not been previously reported.

## Case presentation

**Fig. 1:**
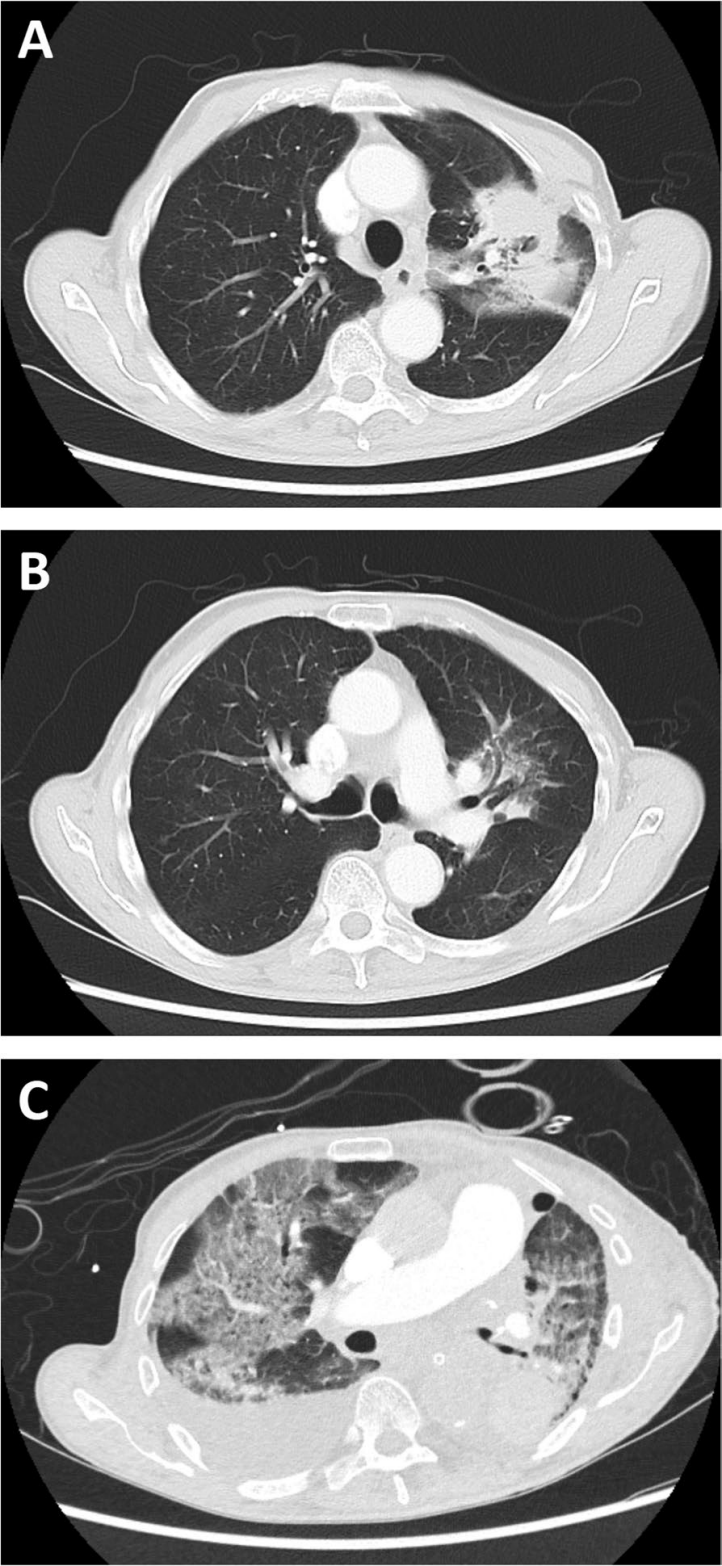
**A-C** Preoperative (**A**, **B**) and postoperative (**C**) transverse CT images of the thorax

A 78-year-old male patient presented for resection of a cT4NxM0 left upper lobe lung adenocarcinoma. His past medical history was significant for hypertension, hyperlipidemia, previous smoking history, ulcerative colitis and proctitis for which he was receiving Sulphasalazine. The patient did not receive corticosteroids or neoadjuvant chemo- or radiotherapy. Preoperative spirometry was unremarkable. Preoperative computerised tomography (CT) scan of the chest revealed a left upper lobe mass with adjacent pleural tethering and consolidative changes proximate to the left mainstem bronchus (LMSB). As multiple small calcified lymph nodes were seen in the right hilar and subcarinal regions (Fig. 1A, B), the patient was planned for mediastinoscopy and lymph node sampling, followed by left upper lobe wedge resection should frozen section examination of the right hilar and subcarinal lymph nodes return negative for malignancy. The left mainstem bronchus measured 13.1 mm (anteroposterior) by 14.0 mm (craniocaudal) on the preoperative CT. Measurements were taken 2 cm distal to the carina, perpendicular to the axis of the bifurcation [3]

General anesthesia was induced with propofol, remifentanil and atracurium. After induction, a single lumen tube (single-use polyvinyl chloride endotracheal tube 7.5 mm internal diameter) was inserted under direct laryngoscopy on first pass and secured at 23 cm at the lips. Endotracheal tube introducers were not used. Using a standard anesthetic breathing circuit and anesthetic machine, positive pressure ventilation was instituted with pressure-control mode with a peak airway pressure of 20cmH_2_O and positive end expiratory pressure of 4cmH_2_O, achieving a tidal volume of 8 mL.kg^−1^ in a 2L.min^−1^ air:oxygen mix. Maintenance of anesthesia was performed with total intravenous anesthesia of propofol and remifentanil, titrating the effect site concentrations to achieve an appropriate depth of anesthesia according to bispectral index monitor. The patient was paralysed with an atracurium infusion.

Mediastinoscopy was performed in supine position via a suprasternal incision, with dissection along the pre-tracheal fascia. The mediastinal lymph nodes frozen section returned negative for malignancy, and surgery proceeded to resection of the left upper lobe lesion via a left open thoracotomy. With the patient still in supine position, and using a bronchoscope (Ambu® aScope™ 4 Broncho Slim 3.8 mm outer diameter), a 9Fr Fuji Uniblocker® was inserted into the LMSB with the balloon deflated. The balloon was inflated with air incrementally under bronchoscopic guidance to a volume of 7 mL to achieve lung separation – within the manufacturer-specified maximum volume of 8 mL. The volume of air required was taken note of and the balloon was then deflated before turning the patient to the right lateral decubitus position. After final patient positioning, bronchoscopy was again used to confirm the position of the EBB and the balloon inflated to the required volume for lung separation. No obvious irregularity or compression of the LMSB was noted on bronchoscopy. There was no sign of blood before, during and after balloon inflation. Initial EBB balloon pressure measured via the pilot balloon was 31cmH_2_O. A Portex® cuff inflator pressure gauge was used intraoperatively for balloon pressure measurement. The EBB was not manipulated following placement, and there was no patient coughing throughout surgery. One-lung ventilation was instituted using pressure control with a peak airway pressure of 24cmH_2_O and positive end expiratory pressure of 8cmH_2_O. An air leak of approximately 100-150 mL per breath was detectable following inflation of EBB balloon, but this was managed with an increase in gas flows to 4L.min^−1^, sufficient to prevent collapse of the ventilator bellow and to achieve a tidal volume of 6 mL.kg^−1^. There was no desaturation, abnormal capnography or abnormal airway pressure or flow curves.

**Fig. 2:**
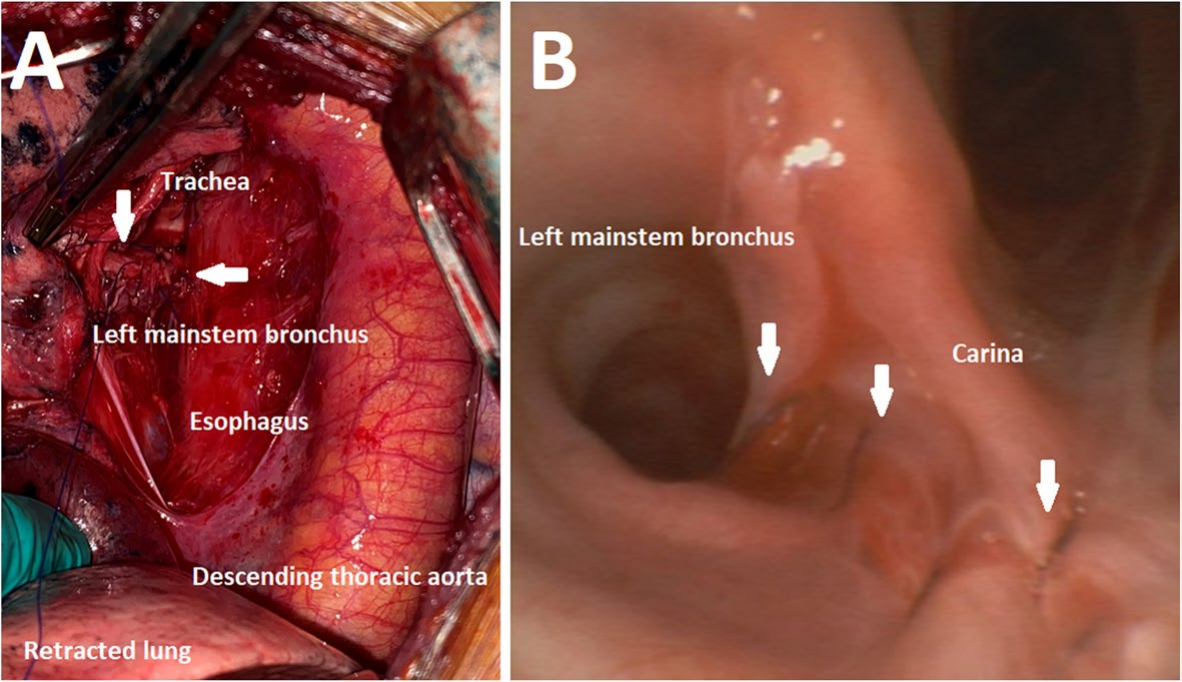
**A-B** Intraoperative photographs demonstrating the site of the perforation within the surgical field (**A**) and on bronchoscopy (**B**). Arrows delineate the location of the left mainstem bronchus rupture

A large left upper lobe tumour (3.3 cm × 5.8 cm) with pleural puckering and dense adhesions between the left hilar tissues was found intraoperatively. During surgical dissection around the LMSB, multiple air pockets with air-trapping were noted between the mediastinal pleura and mediastinal organs. A rupture was found in the posterior wall of the LMSB starting just below the carina and extending 7 cm distally (Fig. 2A, B), with the EBB balloon seen just beneath the peribronchial tissue. A large volume air leak was noted immediately during surgical dissection of the surrounding tissue with complete collapse of the ventilator bellow. The EBB was immediately deflated and removed, and the ETT guided into the right mainstem bronchus using a fiberoptic bronchoscope and the ETT balloon inflated to a pressure of 28cmH_2_O. Thus, one-lung ventilation was achieved with right endobronchial intubation.

The LMSB was repaired with 3/0 Polydioxanone (PDS) sutures and tagged to the esophageal wall posteriorly. The ETT was then withdrawn into the trachea under bronchoscopic guidance following repair of the LMSB. The left hemithorax was then irrigated with povidone iodine and saline, and no air leak was detected with a Valsalva maneuver of 40 cmH_2_O. The Valsalva maneuver was performed by switching the ventilator to manual ventilation and the adjustable pressure-limiting valve closed to 40 cmH_2_O. Fresh gas flow was increased and the breathing circuit bag squeezed for 15 seconds to generate the needed Valsalva maneuver pressure. 40 cmH_2_O was used at the surgeon’s request. No subcutaneous emphysema was present on clinical examination.

Throughout the operation, the patient was relatively stable hemodynamically. There was a slight drop in blood pressure during initiation of one-lung ventilation but this was resolved with boluses of phenylephrine and ephedrine. There was no significant hypoxia during one-lung ventilation and the lowest saturation recorded was 96%.

The patient was extubated uneventfully at the conclusion of surgery and transferred to the intensive care unit. Broad-spectrum antimicrobial cover with Piperacillin-Tazobactam was initiated empirically. However, on the 3^rd^ postoperative day, the patient developed altered mental status and severe bilateral pneumonia, worse on the right – the dependent side intraoperatively (Fig. 1C). This required subsequent reintubation and positive pressure ventilation. Post-operative bronchoscopy and CT revealed the LMSB repair to be intact. The postoperative course was subsequently complicated by acute respiratory distress syndrome, and a left lower lobar pulmonary embolism. The patient received a total of 67 days of positive pressure ventilation, of which 2 days were in the prone position. Unfortunately, the in-hospital stay was further complicated by multi-organ dysfunction, leading to the eventual demise of the patient.

## Discussion and conclusion

Tracheobronchial rupture is a rare, but serious and potentially fatal complication of airway instrumentation that has been reported with the use of endotracheal tube introducers, DLTs, and EBBs [2–5]. The incidence of post-intubation tracheobronchial rupture is difficult to estimate due to its rarity, but is estimated to occur in 1:20,000 to 1:75,000 intubations [6, 7]. The estimated incidence following DLT insertions is 0.05% to 0.19% [6].

Risk factors for post-intubation tracheobronchial rupture have been previously described, broadly divided into mechanical and anatomical factors as summarised in Table 1 [2, 7–12]. In this unfortunate case, advanced age, tumour-related inflammation and adherent, friable soft tissue surrounding the LMSB were the only predisposing risk factors. We opine that (1) poor tissue quality, compounded by (2) trauma by the preformed distal tip of the Fuji Uniblocker, and (3) pressure exerted by the endobronchial blocker balloon on the LMSB, could have led to bronchial rupture.

**Table 1:** Risk factors for post-intubation tracheobronchial rupture [2, 6–10, 13]

Mechanical
Multiple attempts
Operator inexperience
ETT introducers that protrude beyond the tube
Emergency intubation
Cuff overinflation
Incorrect tube positioning
ETT manipulation without cuff deflation
Inappropriate tube size
Dual-lumen tube use
Vigorous coughing
Movement of the head and neck while intubated
Dislodgment or tube movement
Anatomical
Congenital tracheobronchial abnormalities
Weakness of the pars membranosa
Chronic obstructive pulmonary disease
Inflammatory lesions of the tracheobronchial tree
Diseases altering the tracheobronchial tree position or anatomy (e.g. lymph nodes, tumours)
Chronic steroid use
Radiotherapy
Poor biological condition
Advanced age
Height < 165 cm
Female gender

*Abbreviations*: *ETT* Endotracheal tube

The Fuji Uniblocker® is an EBB incorporating a steel mesh polyurethane-coated shaft and preformed distal curve designed to facilitate torque control and direction into the target bronchus. The possibility of a preformed endobronchial device causing bronchial perforation is not far-fetched despite its flexibility, given previous reports of bronchial rupture associated with the use of gum elastic bougies and EBBs [2, 4]. This appears to parallel the only reported EBB-related bronchus rupture in literature – a bronchus perforation by EZ-Blocker™ (Rusch®, Anaesthet-IQ, the Netherlands), an EBB with a preformed Y-shaped distal end designed to mirror the carina [2], in a patient who had received neoadjuvant chemoradiation. In addition, as seen from Fig. 2B, the bronchial rupture occurred in the pars membranacea of the LMSB—a region of relative weakness compared to the cartilaginous part of the bronchus. It is possible that a bronchial rupture could have been prevented if the preformed distal tip of the Fuji Uniblocker® was not turned towards this weak spot (i.e. turning the EBB no more than 90 degrees to the left).

While a pre-existing bronchial defect or iatrogenic trauma during mediastinoscopy are potential etiologies of bronchial rupture, there were no features to suggest these. For example, there was no pneumomediastinum or pneumothorax on preoperative imaging to suggest pre-existing bronchial defect, nor was there significant air leak during ventilation throughout mediastinoscopy to suggest iatrogenic trauma. Furthermore, there was no visualisation of a LMSB lesion or any sign of blood on fiberoptic bronchoscopy during initial EBB placement. Nevertheless, it is still possible that partial thickness tear of the LMSB may have occurred during mediastinoscopy, which contributed to, or resulted in, the subsequent bronchial rupture. The presence of an air leak following inflation of the EBB balloon should be seriously considered as a herald of tracheobronchial injury, as in this case. However, air leak prior to surgical dissection was also likely limited by adherent peribronchial tissue around the rupture site.

High balloon pressures may compromise mucosal blood flow leading to ischemia and mucosal injury, and balloon pressures ≤ 30 cmH_2_O are usually recommended [5, 11]. However, recommendations for endobronchial balloon pressures are less clear and are largely extrapolated from those used for endotracheal tubes, despite the anatomical differences between the trachea and mainstem bronchi [14]. Balloon pressures far exceeding 30 cmH_2_O have been reported without complications [15]. Previous work has demonstrated that balloon pressure differs significantly from pressure exerted on the bronchial wall, although the direction and magnitude of the difference is contentious [13, 14]. One study reported that only a fraction (10 to 20%) of balloon pressure is transmitted to the bronchial wall, and even at much higher balloon pressures, transmitted pressures remain below recommended values for mucosal ischemia prevention [13, 15]. Thus, the argument of whether a balloon pressure of 31cmH_2_O could have ruptured the mainstem bronchus in the absence of pre-existing pathology remains to be tested. However, we opine that the bronchial balloon pressure might have been falsely low in the context of a transmural tear; it is possible that in the context of a pre-existing mural defect, or as a bronchial tear develops around the balloon, progression of the tear and opening up of the bronchial wall reduces pressure on the balloon and hence, measured pressure, providing false reassurance.

Close monitoring of balloon pressure measurements during surgery may be desirable, and the concept of an instrumented balloon allowing measurement of balloon pressure exerted on the airway mucosa has been suggested [14]. However, its utility as a reflection of transmural pressure is uncertain [13, 14]. The required balloon volume and pressure is a delicate balance between preventing air leak or contamination and preventing mucosal ischemia. Additionally, patients requiring lung separation usually include those with pre-existing pulmonary pathology who may have poorer lung compliance and thus require higher airway pressures during mechanical ventilation, which may be further compounded by dynamic changes under general anesthesia.

Compared to the paucity of reports in EBBs, most published reports of tracheobronchial injury have been described in DLTs which have a larger diameter and are more rigid, possibly predisposing to a higher risk of airway complications [1]. Several reports of tracheobronchial injury caused by DLTs have been published [12, 16–18]. Most commonly, tracheobronchial rupture occurred as a result of inappropriately large DLT sizes, while other purported causes of mainstem bronchus rupture include balloon overinflation and previous irradiation with vulnerable airway tissue. In hindsight, for such a case of a large left upper lobe mass with features predicting bronchial involvement or invasion, it may be prudent to consider a right-sided DLT for lung separation to avoid instrumenting the LMSB.

Tracheobronchial rupture is a major event that requires prompt recognition and management. In this case, the bronchial rupture was recognised simultaneously by both surgeons and anesthetists – the EBB balloon was unexpectedly visualised through the LMSB, and there was a sudden fall in delivered tidal volume, coupled with complete collapse of the ventilator bellow, signifying a large volume air leak. A tracheobronchial rupture might manifest as difficulty in establishing ventilation with abnormally high fresh gas flows to prevent ventilator bellow collapse (which was also present early in this case), desaturation, abnormal capnography waveforms, and decreased breath sounds or chest rise prior to one lung ventilation. A leak in the anesthesia breathing circuit is an important differential diagnosis that should be quickly excluded by visual identification of disconnections, and manual ventilation via the reservoir bag or an alternative self-inflating bag system. Tracheobronchial rupture should be considered if a leak persists after all mechanical components have been checked. Bronchoscopic evaluation should be performed and the airway inspected thoroughly. The essentials of anesthetic management in a tracheobronchial rupture is ensuring adequate ventilation and protection of the airway. If inhalational agents are used, they should be stopped and switched to total intravenous anesthesia. The injured airway would need to be isolated to facilitate surgical repair, and in this case, this was achieved quickly with removal of the EBB and direction of the single-lumen ETT into the contralateral lung.

Lastly, it is interesting that post-operatively, pneumonia was worse on the dependent side. While it is possible that the bronchial rupture, which extended proximally to the level of the carina, could have allowed contamination of the right lung, other post-surgical events such as altered mental status leading to pulmonary aspiration, could also have contributed to this finding.

In conclusion, this case is a reminder that clinicians should be cognisant that bronchial rupture is a rare but potential complication of EBBs that can carry significant morbidity. Its use is a serious process and placement needs to be handled with exceptional care, particularly in patients who may have compromised lung tissues such as from surrounding tumour involvement, soft tissue radionecrosis from radiotherapy, or connective tissue disease. Direct bronchoscopic visualisation should be used during insertion, and the volume and pressure of the balloon kept to the minimum required to prevent air leak. The presence of an air leak should warrant consideration of bronchial rupture as one of the differential diagnoses, as should the finding of unexpected air pockets within the mediastinum.

## Supplementary Information

**Additional file 1**. Fuji Uniblocker® (Fuji Systems Corporation, Japan).

## Data Availability

Not applicable.

## Abbreviations

DLT: Double lumen tube
EBB: Endobronchial blocker
SLT: Single lumen tube
CT: Computerised tomography
LMSB: Left mainstem bronchus
ETT: Endotracheal tube
PDS: Polydioxanone
