# Supplementary figures and images for: Bronchial rupture following endobronchial blocker placement: a case report of a rare, unfortunate complication
Source: BMC Anesthesiol. 2021 Aug 30;21:208. doi: 10.1186/s12871-021-01430-6 (PMC8404020; doi:10.1186/s12871-021-01430-6)

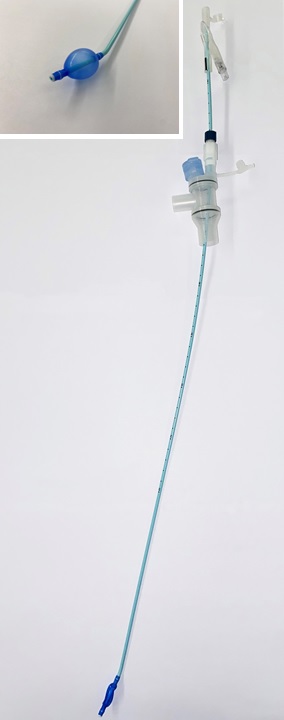

Supplement: Supplementary file 1 — Additional file 1. Fuji Uniblocker® (Fuji Systems Corporation, Japan). [file 12871_2021_1430_MOESM1_ESM.jpg]
